# Evidence for a trade-off between growth rate and xylem cavitation resistance in *Callitris rhomboidea*

**DOI:** 10.1093/treephys/tpad037

**Published:** 2023-03-22

**Authors:** Kate M Johnson, Timothy J Brodribb

**Affiliations:** Biological Sciences, School of Natural Sciences, University of Tasmania, College Road, Hobart 7001, Australia; Biological Sciences, School of Natural Sciences, University of Tasmania, College Road, Hobart 7001, Australia

**Keywords:** conifer, drought, optical technique, plasticity, variation, vulnerability

## Abstract

The ideal plant water transport system is one that features high efficiency and resistance to drought-induced damage (xylem cavitation), however, species rarely possess both. This may be explained by trade-offs between traits, yet thus far, no proposed trade-off has offered a universal explanation for the lack of water transport systems that are both highly drought-resistant and highly efficient. Here, we find evidence for a new trade-off, between growth rate and resistance to xylem cavitation, in the canopies of a drought-resistant tree species (*Callitris rhomboidea*). Wide variation in cavitation resistance (P50) was found in distal branch tips (<2 mm in diameter), converging to low variation in P50 in larger diameter stems (>2 mm). We found a significant correlation between cavitation resistance and distal branchlet internode length across branch tips in *C. rhomboidea* canopies. Branchlets with long internodes (8 mm or longer) were significantly more vulnerable to drought-induced xylem cavitation than shorter internodes (4 mm or shorter). This suggests that varying growth rates, leading to differences in internode length, drive differences in cavitation resistance in *C. rhomboidea* trees. The only distinct anatomical difference found between internodes was the pith size, with the average pith to xylem area in long internodes being five times greater than in short internodes. Understanding whether this trade-off exists within and between species will help us to uncover what drives and limits drought resistance across the world’s flora.

## Introduction

By jeopardizing the continuous supply of water to the leaves, acute water deficit leads to tissue damage and death in plants (Brodribb et al. 2021, Johnson et al. 2021). Drought-stress imposes increasing tension on the water columns in the xylem, which can cause them to break in a process called xylem cavitation (Tyree and Sperry 1988). The resulting air bubbles (embolisms) block water transport and accumulate during drought, eventually leading to plant death (Lewis 1988, Tyree and Sperry 1988, 1989). When viewed in isolation, it would seem that this intrinsic vulnerability in the capacity of plants to survive drought could be easily solved by producing xylem that could resist cavitation; however, given that higher xylem water transport efficiency is associated with higher photosynthetic capacity (Brodribb and Feild 2000, Hubbard et al. 2001), the ideal water transport system is one that is both efficient and resistant to cavitation (Sperry 2003, Hemsley and Poole 2004, Gleason et al. 2016), traits which species rarely possess simultaneously. Xylem conductivity (efficiency of water flow) and vulnerability to cavitation vary widely among species, often correlated with climatic and ecological niches (Blackman et al. 2012, Choat et al. 2012, Brodribb et al. 2014, Skelton et al. 2015). This suggests that these traits are adaptive but that greater development of either cavitation resistance or flow capacity incurs a significant cost to plants.

It has been hypothesized that a direct trade-off exists between xylem cavitation resistance (commonly referred to as ‘safety’) and efficient water flow (efficiency) in plants. This theory suggests that xylem networks cannot simultaneously possess high conductivity to water and high resistance to cavitation (Sperry 2003, Hemsley and Poole 2004, Gleason et al. 2016). The underlying idea is that conduits with larger diameters transport water more efficiently, as the efficiency of water transport through a pipe (such as a xylem conduit) increases to the fourth power of its diameter, as stated by the Hagen–Poiseuille law (Carlquist 1966, Tyree et al. 1994, Comstock and Sperry 2000). Yet, these larger diameters leave conduits more vulnerable to embolism during freeze–thaw, and possibly during drought (Carlquist 1966, Tyree et al. 1994). In conifers, evidence suggests that the pit aperture in the xylem walls governs resistance to cavitation and, likely, efficiency of water flow. A greater degree of overlap between the pit covering (torus) and the pit itself has been linked to greater resistance to cavitation (Bouche et al. 2014). Yet, achieving greater overlap by reducing the pit aperture may also lead to lower efficiency of water transport. No pattern has been found between conduit diameter and vulnerability to cavitation in conifers (Bouche et al. 2014), and support for this relationship is equivocal in vessel-bearing species (Tyree et al. 1994, Lens et al. 2022). This may contribute to the ambiguity of the evidence supporting a trade-off between xylem safety and efficiency, as found by Gleason et al. (2016), who noted that >50% of studies on this topic did not find evidence of this trade-off.

Variation in xylem vulnerability within and between plants presents an excellent tool to investigate possible trade-offs associated with changes in xylem character. Considerable variation in vulnerability to cavitation has been found in leaves and small branchlets within individual canopies of *Persea americana Mill.* (Cardoso et al. 2020), *Olea europa L.* (Rodriguez-Dominguez et al. 2018) and *Callitris rhomboidea R.br* (Johnson et al. 2021). Based on the water potential at which 50% of the xylem rendered non-functional due to xylem embolism (P50; a widely used metric for comparing cavitation vulnerability), the variation found in the canopy of *C. rhomboidea* was particularly high. The range in P50s among individuals of this species of −1.4 to −9.8 MPa was found to approach the range of P50s found in an analysis of 384 angiosperms and 96 gymnosperms (<−1 to −14 MPa, Choat et al. 2012). This extreme case of apparent plasticity provides the ideal study system to investigate whether there may be other functional trade-offs associated with variation in xylem cavitation vulnerability.

A potential trade-off of particular interest is between growth rate and vulnerability to cavitation. Research by Roskilly et al. (2019) found a relationship between growth rate and longevity, where younger trees were faster growing than older trees of the same species. These trees also showed differences in xylem anatomy, with xylem characteristics indicative of greater cavitation resistance in the older trees (Roskilly et al. 2019). This is possible evidence of a growth rate—vulnerability trade-off, where slower growth and greater longevity may be linked to a greater ability to survive drought though greater resistance to xylem cavitation.

Here, we test the hypothesis that a trade-off between branch growth rate and xylem cavitation resistance is driving the extensive variation found in vulnerability to cavitation in the canopies of *C. rhomboidea* (Johnson et al. 2021). If a trade-off between growth rate and drought resistance exists in *C. rhomboidea* canopies, we would expect that long internodes (representing fast growth rates) would be more vulnerable to xylem cavitation than short internodes (representing slow growth rates). This would be further supported by differences in xylem or stem anatomy between these groups. Using the optical vulnerability technique (OVT), a relatively new and non-invasive method for monitoring embolism, we (i) investigated whether stem diameter influences vulnerability to cavitation to determine the location of the high variation in P50 previously found in *C. rhomboidea* canopies (Johnson et al. 2021) and (ii) tested for a trade-off between growth rate and vulnerability by quantifying vulnerability in internodes of varying lengths (assumed to reflect differences in local growth rate during internode extension). We then analyzed characteristics of the xylem conduits and the anatomy of long and short internodes using transverse light microscope sections, to seek some anatomical correlation with xylem vulnerability.

By using a combination of plasticity and within-species variation to investigate variation in vulnerability to xylem cavitation, we test whether a new factor (growth rate) may be linked to drought resistance within *C. rhomboidea* canopies. The results provide insights into possible drivers of the variation in drought resistance within this species, among conifers and possibly across other plants.

## Materials and methods

### Plant material

An Australian native tree, *C. rhomboidea*, was chosen as a test species based on its extreme drought resistance, the wealth of literature on this species (Heady et al. 1994, Brodribb and Cochard 2009, Pittermann et al. 2010, Bouche et al. 2014, Larter et al. 2017, Bourbia et al. 2021) and recent findings showing wide variation in cavitation resistance within its canopy (Johnson et al. 2021).

### Stem diameter and vulnerability

To determine the potential effect of stem diameter on vulnerability to cavitation, branches between 50 and 100 cm were cut from a single adult tree of *C. rhomboidea* growing on campus at the University of Tasmania, Hobart (42.8826°S, 147.3257°E). The aim of this experiment was to determine how variation in xylem vulnerability was related to stem diameter within a single genotype. Hence, a large number of samples were taken from a single individual in order to reduce the potentially confounding influence of within-species variation. A total of 3–5 stem segments per branch across seven branches (totalling 25 stem segments) were selected for vulnerability measurements. Cameras (Cavicams; cavicam.co, and microscopes) were placed sequentially along branches, where the largest stem diameters (up to 7 mm) were monitored closer to the cut end and the smallest diameters (down to 0.5 mm) were measured toward the distal branch tips. While internode length was not measured for the samples used in this experiment, all distal branchlet internodes were found to be 8 or <8 mm in this tree.

### Internode length and vulnerability

To investigate the effect of internode length on vulnerability, it was necessary to move beyond the individual tree level in order to capture the largest range of growth rate variation. Thus, branches between 50 and 100 cm in length were collected from five trees grown under a range of conditions: (i) potted glasshouse-grown saplings ~1 year old, (ii) outdoor raised potted trees ~2 years old and (iii) branches from the adult tree of *C. rhomboidea* mentioned above. Material was sourced from trees grown under a variety of conditions to maximize the range of growth rates (therefore internode lengths) that we were able to sample. However, internodes of varying lengths were measured from each of these individuals, in order to extricate the effects of individual variation from those produced by growth rate. Therefore, here, we investigate both phenotypic variation within individuals and variation between individuals of *C. rhomboidea*.

The idea that internode length reflects growth rate is supported by research showing that trees in Cupressaceae (such as *C. rhomboidea*) exhibit year-round growth with seasonal variation in internode length and a ‘fixed’ growth habit, meaning that an increase in growth rate cannot be reflected in an increase in the number of internodes, only in internode length (Wilson 2000). Grosfeld and Barthelemy (2004) found that internode lengths varied seasonally, with those grown in late summer–early spring found to be shorter than those grown in mid-late spring in three members of *Cupressaceae*. The rate of elongation-growth in plants is also known to be strongly driven by environmental factors such as temperature (Herman 1956, Zobel 1983) and water availability (Harry 1987).

Vulnerability was measured in 3–5 internodes per branch in 7 branches, resulting in a total of 21 distal branchlet internodes. These internodes ranged from 2 to 20 mm in length and 1 to 2 mm in diameter. All measured internodes were within 20–100 mm of the branch tip, and distance to the branch tip did not have a significant effect on vulnerability (ANOVA; *P* > 0.05) with similar P50s regardless of distance to the tip (see Figure S1 available as Supplementary data at *Tree Physiology* Online). We focused on distal branch tips as this is where tissue death is often first observed (Brodribb and Cochard 2009, Brodribb et al. 2021, Johnson et al. 2021).

### Quantifying xylem vulnerability

Xylem embolism was monitored using the OVT (Brodribb et al. 2016, 2017). A sharp razor was used to remove photosynthetic (leaf) tissue from one side of the stems and the distal internodes to expose the xylem (ensuring that at least 50% of the phloem remained intact). A clear hydrogel (Tensive Gel; Parker Laboratories Inc., Fairfield, NJ, USA) was applied to reduce evaporative water loss and improve light transmission (Johnson et al. 2021). A combination of Raspberry Pi-based time-lapse cameras with 30× magnification and LED illumination (Cavicams; cavicam.co), and a Leica light microscope (Leica model DFC450C Nikkor 60 mm Macro lens) were used to visualize cavitation in the stems and distal internodes. Images of the xylem were captured every 3 min as the branches dried, while stem water potential was measured concurrently using a stem psychrometer (ICT PSY Armidale, NSW, Australia).

### Image analysis

An image subtraction procedure was performed in ImageJ (Schindelin et al. 2012) to reveal the changes in light transmission, which occur when xylem rapidly transitions from water filled and functional to air-filled and non-functional due to cavitation (Brodribb et al. 2016, Johnson et al. 2021). Other gradual changes associated with tissue desiccation during dehydration were filtered out to highlight only the rapid changes in pixel intensity associated with cavitation events (see http://www.opensourceov.org/ for more details). Embolized pixels were then expressed as a percentage of the total embolized pixel area and plotted against branch water potential to determine vulnerability to cavitation.

### Internode and xylem anatomy

Tracheid area and diameter were calculated using transverse light microscope sections from 11 ‘short’ internodes (lengths of 4 mm and shorter) and 10 long internodes (lengths of 8 mm and greater) sampled from the same trees, which were used for vulnerability calculations. ImageJ was used to threshold images and highlight the tracheids as ellipses. Tracheid diameters and areas were extracted from the ellipses as described in Johnson et al. (2021) and the average values were determined for each transverse microscope section, and then collectively for all short and long internodes.

Transverse light microscope sections were also used to calculate the relative pith and xylem areas within internodes. The pith area was traced using the ‘line drawing tool’ in ImageJ. The features outside this region were then cleared to white using the ‘clear outside’ function. The traced region was ‘filled’ and thresholded to allow the area to be calculated. To determine xylem area, the area encompassing both xylem and pith was traced and filled to calculate the total area, from which the previously calculated pith area was subtracted.

### Shrinkage

To test whether the timing or extent of physical tissue-deformation during drying was different between internode length classes, the shrinkage (reduction in width) of long (8 mm or longer) and short (4 mm or shorter) internodes sourced from trees exposed to different growing conditions during dehydration was determined from the same images captured during embolism monitoring. ImageJ was used to measure the initial (fully hydrated) width of the internodes (from the first image captured) and this was compared with the internode width at both −3 and −5 MPa, as these water potentials were shown to be associated with significant cavitation.

### Xylem ultrastructure: pit properties

Due to the laborious nature of measuring pit-level characters, a subsample of four of the internodes measured for vulnerability to cavitation, which were sourced from trees grown under glasshouse conditions, were imaged using a field emission scanning electron microscope (FESEM) in order to investigate xylem ultrastructure, specifically inter-tracheid pit properties. These four internodes (comprising three long and one short internode, which were successfully sampled and prepared) represented the extremes of internode length and vulnerability and were therefore chosen as they were determined to present the greatest chance of finding differences between samples. Post vulnerability measurements, internodes were snap-frozen in liquid nitrogen, then freeze-dried overnight as described in Pittermann et al. (2010). Forceps were placed at the center of the samples to split the wood longitudinally. This was to split the samples along the weakest plane (the gap between conduit walls) to reveal the pits that connect tracheids through their outer walls. Images of 10–20 margos per internode were captured and the pit aperture area and diameter along with the density of papillae were quantified using ImageJ, where margo surfaces were categorized according to the density of papillae (0–50, 50–100, 100–300 and >300).

### Data analysis

Statistical significance was tested in R v.4.0. (RStudio Team 2016). Vulnerability measurements were compared for internodes from different trees using linear mixed effect models in Rstudio v.4.0.2 (RStudio Team 2016), with P50 or P88 as the response and tree ‘ID’ and replicate (branch) as fixed effects as described in Johnson et al. 2021. We used the *lmer* function in the *lme4* R package (Bates et al. 2015), and to account for repeated measures (pseudoreplication), we included tree ID as a random effect to satisfy the assumptions of a linear model (Harrison et al. 2018). To test for differences in P50s and P88s within and among trees, we used the ANOVA function in the car R package (Fox and Weisberg 2019). Plots were constructed using SIGMAPLOT v.12.5 (Systat Software 2003) and R v.4.0.

## Results

### Vulnerability and stem diameter

**Figure 1:**
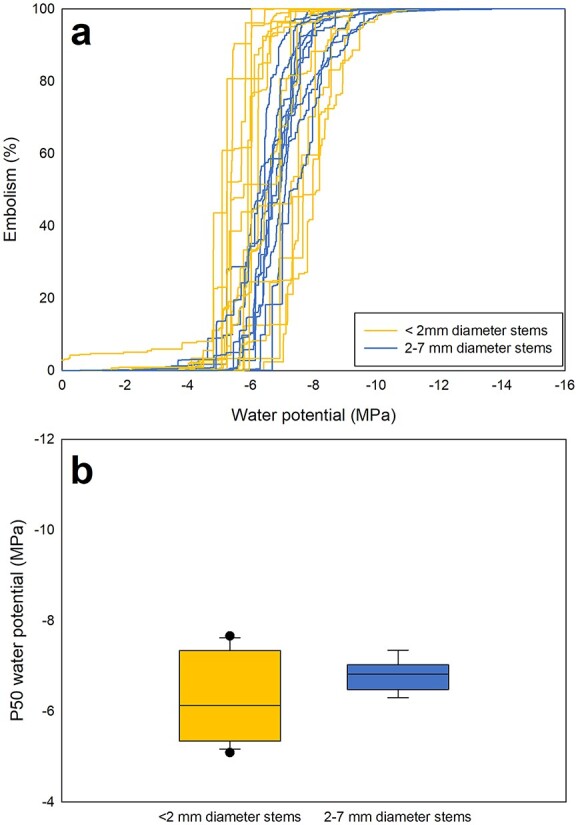
Vulnerability curves (a) and median P50s (b) for branches of *C. rhomboidea* with different diameters measured from a single adult tree growing on campus at the University of Tasmania in Hobart.

While there was no significant difference in mean P50s of stems with diameters <2 mm and those 2–7 mm from branches of an adult *C. rhomboidea* tree (−6.3 and −6.7 MPa, respectively; ANOVA, *P* > 0.05), the smaller diameter class showed significantly higher standard deviation and variance in P50 than the 2–7 mm stems (SD = 0.9 MPa in stems <2 mm compared with SD = 0.3 MPa in stems 2–7 mm, F18,8, *P* < 0.01, Figure 1). The grouping of stems into diameter classes (<2 mm and 2–7 mm) reflects these distinct differences in variation and also relates to the position of the tissue, with the smallest diameters occurring out toward the tips of the branchlets and the larger diameters occurring further upstream. The average P50s of both diameter classes were similar to that previously reported for small branches of *C. rhomboidea* (−6 MPa, Brodribb and Cochard 2009).

### Vulnerability and internode length

**Figure 2:**
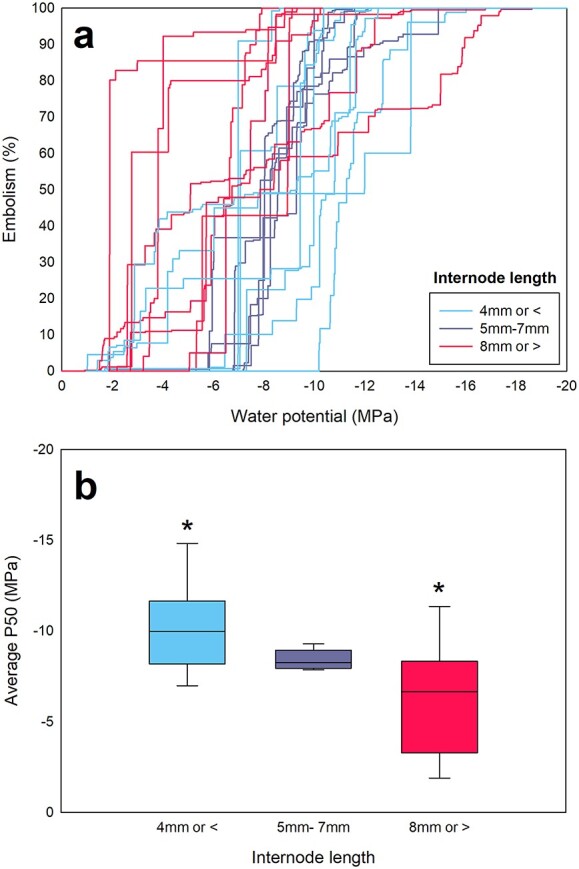
Vulnerability curves (a) and median P50s (b) for distal branchlet internodes with different lengths sampled from *C. rhomboidea* trees grown under a range of conditions.

The P50s of distal branchlet internodes (all with diameters of 2 mm or less, with internode lengths ranging from 2 to 20 mm) were highly variable (−1.89 to −14.82 MPa, Figure 2). The mean P50 of short internodes (4 mm or less) was significantly more negative (−10.19 MPa ± 0.81) than the mean P50 (−6.12 MPa ± 1.01) for long internodes (8 mm or greater, Figure 2b; ANOVA, *P* < 0.05). Plotting P50 against internode length showed grouping of internode P50 by size across trees (see Figure S2 available as Supplementary data at *Tree Physiology* Online). A linear mixed-effects model followed by an ANOVA found no significant difference in the average P50s or P88s of internodes from different trees (*P* > 0.05).

**Figure 3:**
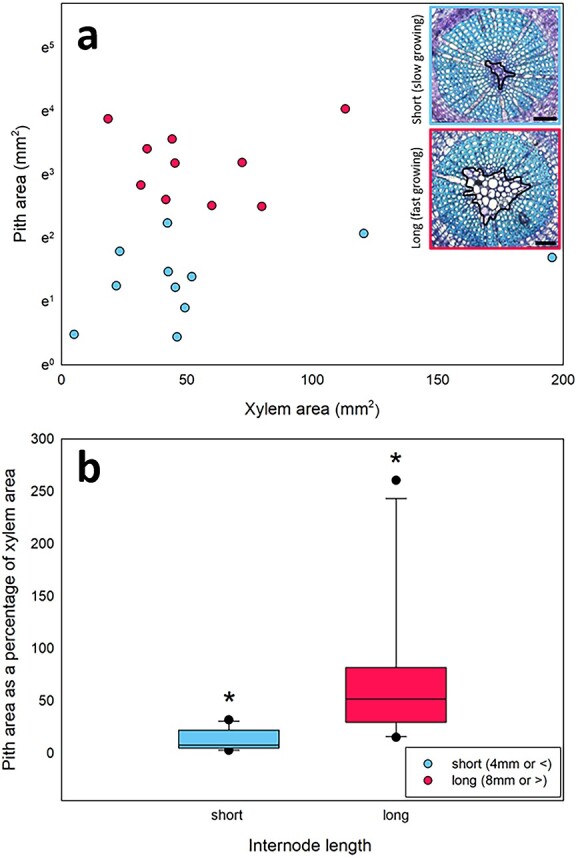
Xylem area against pith area (a) and pith area as a percentage of xylem area (b) for long distal branchlet internodes (8 mm or >^*^, pink) and short distal branchlet internodes (4 or <4 mm, blue).

The pith to xylem ratio was consistently larger in long distal branchlet internodes (8 mm or greater in length) than in short internodes (4 mm or less in length, Figure 3a). While the xylem area was similar in most transverse microscope sections (<100 mm^2^), pith areas were consistently larger in long internodes, compared with short internodes (Figure 3a). On average, pith area, as a percentage of xylem area, was more than five times greater in long internodes (68.8%, compared with 12.6% in short internodes) and was significantly different between these groups (ANOVA, *P* < 0.05; Figure 3b).

### Anatomical associations

Both mean tracheid diameter and area were similar across transverse microscope sections of internodes regardless of length (ANOVA, *P* > 0.05; Figure S3 available as Supplementary data at *Tree Physiology* Online). This consistency was found despite the differences in vulnerability to xylem cavitation between the longest (fastest growing) and shortest (slowest growing) internodes (Figure 2).

SEM imaging showed high variability in the density of papillae present on the outer surface of inter-tracheid bordered pits across all internodes, with no correlation found with internode length (ANOVA, *P* > 0.05; Figure S4 available as Supplementary data at *Tree Physiology* Online). Analysis of pit size from SEM imaging revealed that the average pit aperture area of the most vulnerable internode measured here (P50: −1.89 MPa) was significantly (*P* > 0.00005) larger than the three remaining internodes (see Figure S5 available as Supplementary data at *Tree Physiology* Online), while there were no significance differences in pit apertures found between the remaining internodes.

The extent of shrinkage during dehydration was found to be similar across long (8 mm or greater) and short (4 mm or less) internodes. There was no significant difference in the extent of shrinkage at −3 MPa (short: 7.2%, long: 8.3%) or at −5 MPa (short: 11.7%, long: 13.8%, ANOVA, *P* > 0.05; Figure S6 available as Supplementary data at *Tree Physiology* Online).

## Discussion

Here, we present evidence for a trade-off between the growth rate of branchlets and resistance to xylem cavitation in *C. rhomboidea* canopies. The large variation in P50 previously observed across the canopies of this species (Johnson et al. 2021) was found to be confined to small-diameter distal branchlets (<2 mm). Within these distal branchlets, significant correlation was found between P50 and internode length. Branchlets with internodes 8 mm or longer were much more vulnerable to drought-induced xylem cavitation than shorter internodes (4 mm or shorter), supporting the idea that faster growth rates are associated with more vulnerable xylem and vice versa. The only significant anatomical difference associated with these large differences in P50 was a substantially higher pith to xylem ratio in the longer (faster growing) internodes.

### P50 and stem diameter

The finding that the P50 is less variable in larger diameter stems (>2 mm) than those <2 mm has important practical implications for measuring vulnerability to xylem cavitation in stem tissue (Figure 1). The greater consistency in P50 within larger diameter stems (2–7 mm) is likely to be due to the ‘smoothing’ effect of a larger sample size of conduits, as depicted in the vulnerability curves for the 2–7 mm sizes class in Figure 1. This is reassuring for those interested in characterizing stem vulnerability for a species. Our results suggest that consistent P50s can be obtained for an individual plant (or species) as long as branch thickness is sufficiently large to integrate several seasons of growth. Finding consistent P50s in larger diameter stems supports the reliability of data from the wealth of studies, which quantify stem vulnerability using stems with diameters >2 mm (Delzon et al. 2010, Choat et al. 2012, 2016, Brodribb et al. 2017, Skelton et al. 2019, Smith-Martin et al. 2020). In fact, it appears that stems >2 mm have been used in all previous studies of stem vulnerability. The basipetal decrease in the variability in vulnerability to cavitation found here also suggests that the large variation in *C. rhomboidea* vulnerability found here and in Johnson et al. (2021) is confined to the distal branch tips.

While a larger number of xylem conduits likely explains the lower variability in P50s found in larger diameter stems, we do not think that a smaller number of conduits in small diameter stems and internodes is the only factor explaining the higher variability in their P50s. The suggestion that variation in P50 is determined by the sample size of conduits alone implies that individual conduit vulnerabilities are randomly distributed within stems. Instead, we propose that assuming environmental conditions have a strong effect on xylem vulnerability, younger, smaller diameter stems and internodes represent short periods of growth with xylem grown under similar conditions that have similar vulnerabilities as a result. We propose that different seasonal conditions at the time of growth in different young branches/internodes (i.e., in samples with diameters of <2 mm) would lead to the greater variation observed between these samples, which is supported by research showing that season affects internode length (Grosfeld and Barthelemy 2004). In contrast, stems with diameters >2 mm would contain xylem produced under a range of conditions, leading to more consistent P50s that represent an average of the variable conditions experienced during growth.

The few, large cavitation events, which characterize embolism progression in smaller stems, also point to greater similarity in conduit xylem vulnerability within these stems. The contrast between the pattern of embolism in <2 mm stems and 2–7 mm in Figure 3 is striking, with many cavitation events embolizing 30–60% of conduits in smaller-diameter stems, with most cavitation events in larger-diameter stems embolizing 10% or fewer of the total number of conduits.

It should also be noted that differences in water potential across *C. rhomboidea* canopies may have contributed to the differences we found in branch-tip xylem vulnerability. As water potential was monitored in the main stem only, we do not know how much water potential varied across tree canopies, including within the internodes we monitored. Even if there was some variation in water potential, because cavitation was monitored under drought conditions, well beyond stomatal closure, we would expect water potential to have been relatively consistent between branches within samples. Due to low transpiration rates, water potential divergence would only be expected when xylem was almost completely blocked with embolisms, at which point the measurement of vulnerability is complete. Additionally, if differences in within-canopy water potential were driving the observed differences in internode vulnerability, water potential would have been consistently different between long and short internodes. The finding that there was no significant difference in the shrinkage of long and short internodes (see Figure S4 available as Supplementary data at *Tree Physiology* Online), where shrinkage is known to correlate with water potential (Bourbia et al. 2021, Johnson et al. 2021), points to uniformity in internode water potential. Based on this, we think that it is very unlikely that differences in internode water potentials alone can account for the wide range of xylem vulnerabilities.

### A trade-off between growth rate and cavitation resistance

The correlation between P50 and distal branchlet internode length in the canopies of *C. rhomboidea* suggests that differential growth rates, leading to differences in internode length, may drive the wide variation observed in xylem vulnerability. The idea of a growth rate vs vulnerability trade-off presents an alternative to the well-known ‘safety vs efficiency’ hypothesis for which limited evidence has been found, despite repeated testing (Gleason et al. 2016). The observation that internode length and xylem conduit size are not linked (see Figure S3 available as Supplementary data at *Tree Physiology* Online), combined with the correlation found between internode length and vulnerability (Figure 2), suggests that the relationship between growth rate and vulnerability does not involve conduit size. This is supported by research finding that conduit size and vulnerability are not linked in conifers (Bouche et al. 2014). The evidence presented here, suggesting a trade-off between the resistance of xylem to cavitation and the rate of distal branchlet internode extension, may be driven by differences in growth conditions between internodes.

One possible explanation for the weak correlation between xylem safety and efficiency is that some other traits, linked to hydraulic conductance, may also limit cavitation resistance. For example, it is possible that xylem growth rate affects vulnerability to cavitation. As xylem conduits are dead at maturity, vulnerability to cavitation is likely determined at the time of xylem development (Samuels et al. 2006, Holbrook and Zwieniecki 2011, Bartlett et al. 2016, Lemaire et al. 2021). This means that the conditions at the time of xylem production may drive vulnerability to cavitation, possibly through differences in the rate of xylem expansion.

Research has shown examples of plasticity in vulnerability to cavitation in response to environmental conditions. Within plants, differences in traits associated with drought resistance have been found between tree rings of *Larix decidua* and *Picea abies* in response to summer drought (Bryukhanova and Fonti 2013), and xylem conduits located in close proximity have been shown to be more likely to embolize together (Mrad et al. 2018, 2020, Johnson et al. 2020). It therefore seems likely that xylem produced at the same time, therefore under the same environmental conditions, would have similar vulnerability to cavitation. While the average P50s obtained from small-diameter stems of *C. rhomboidea* in this study and in Brodribb and Cochard 2009 (~−6 MPa) were similar, this is different from the P50 obtained from samples of similar sizes at a field site with low annual rainfall (~−7 MPa, Smith-Martin et al. 2020). The more negative P50 in the dry field site (annual rainfall ~600–700 mm) may be evidence of plasticity, where dry conditions lead to lower growth rates, shorter internodes and more negative P50s.

While plasticity in vulnerability to xylem cavitation has been studied within species, limited research shows evidence of variability in this trait within individual plants (Bryukhanova and Fonti 2013, Johnson et al. 2018, Rodriguez-Dominguez et al. 2018, Cardoso et al. 2020). Although we do not solely focus on plastic variation here, this study is confined to a single species, meaning that the influence of genetic variation is likely limited. The large amount of plastic and within-species variation that we find here in vulnerability to cavitation, internode length and the pith to xylem ratio in the canopy of a single species presents an ideal opportunity to study what may be driving differences in cavitation vulnerability.

### Possible mechanistic explanations for this trade-off

While we find evidence supporting a trade-off between growth rate and xylem vulnerability, we still do not know the mechanistic drivers of the observed variation in vulnerability within *C. rhomboidea* canopies (Johnson et al. 2021). The similarity in xylem area but disparity in pith areas between long and short distal branchlet internodes suggests that variable growth rates lead to differences in the allocation of tissue within branchlet internodes, which is correlated with xylem cavitation resistance. It is possible that the differences in distal branchlet internode construction, leading to variation in pith area, drive the differences in vulnerability in *C. rhomboidea* canopies.

The lack of difference in the shrinkage of the long (fast growing) and short (slow growing) internodes indicates that increased pith area is unlikely to lead to cavitation by stem collapse and consequent deformation of the xylem. However, evidence from 3D scanning of stems indicates that the pith may still play a role in vulnerability to cavitation. Primary xylem located adjacent to the pith were found to be embolized even in hydrated scans of conifer, *Sequoia sempervirens* (Choat et al. 2015). While this could be due to stretch and rupture of these conduits during extension-growth as Choat et al. 2015 suggest, it may also indicate that the pith represents a reservoir of air that could promote cavitation via air seeding (Brodersen et al. 2013*b*). This is supported by research finding that the pith contains far more air than the surrounding xylem (Holbrook et al. 2001, Choat et al. 2010) and evidence that embolism radiates out from this pith, as shown in *Vitis vinifera* (Brodersen et al. 2013*a*, 2013*b*). An increased pith area, equating to a larger reservoir of air, may therefore increase the chance of air embolism in the adjacent xylem that could radiate throughout the stem. While the higher pith to xylem ratio may play a role in increased vulnerability, it is also possible that differences in vulnerability may be driven, at least in part, by differences in the xylem conduits themselves, with origins in processes that lead to their development.

Differential growth rates may be leading to structural differences in the secondary xylem walls, influencing vulnerability to cavitation in distal branchlet internodes. Growth rate has been shown to affect the construction of the xylem walls through differences in the arrangement of the cellulose microfibrils in xylem and fiber walls (Microfibril angle; MFA). Like internode length and xylem vulnerability, MFA has been shown to be influenced by environmental conditions (Barnett and Bonham 2004, Donaldson 2008). Wimmer et al. (2002) linked drought stress to smaller MFA (stiffer tissue) and release from drought stress to an increase in MFA (more elastic xylem walls) in *Eucalyptus nitens*. This suggests that drought may limit growth rate, leading to smaller MFAs and greater stiffness, and that when water is made available, growth rate increases along with MFA and elasticity. Differences in MFA may also directly affect the pits in xylem walls.

As the ‘pits’, the tiny pores through which cavitation occurs are located in the xylem walls, it seems likely that these structures could also be affected by growth-rate-driven differences in xylem construction. Previous research has found that an association between the papillae on xylem pits and rainfall environment in *Callitris* species, with large, nodulated papillae, was found to be associated with species from drier environments (Heady et al. 1994). While the SEM scans we conducted in this experiment did not provide sufficient evidence to support this, this hypothesis warrants further investigation.

While thicker pit membranes are strongly linked with greater cavitation resistance in *Arabidopsis* (Thonglim et al. 2021, 2023), we do not know exactly which aspects of pits control vulnerability in conifers (Delzon et al. 2010). Bouche et al. (2014) found that higher torus (pit covering) to pit-aperture ratios (torus overlap) were associated with greater xylem cavitation resistance across 115 diverse conifer species. The authors also found that conduit diameter and vulnerability were not related, similar to the findings presented here for *C. rhomboidea*, suggesting that conifers are able to increase their xylem resistance to cavitation without reducing hydraulic efficiency (Bouche et al. 2014). While we found some indication that larger pit apertures in xylem walls lead to extreme vulnerability, this needs further investigation.

## Conclusions

Evidence for a trade-off between growth rate and xylem vulnerability in small branches of *C. rhomboidea* provides insights into what may limit drought resistance within the canopy of this species. We speculate that increased pith area, leading to an increased reservoir of air, may lead to a greater chance of cavitation and possibly higher vulnerability in longer (faster growing) internodes. We also suggest that differences in the construction of the xylem due to variation in growth rate (possibly related to differences in MFA driven by the action of microtubules in early xylem development) may influence vulnerability. However, the mechanistic cause of the variation in vulnerability found in this species is still unknown. Here, we lay the foundations to reveal what drives the large plastic and within-species variation in P50 found in the canopies of *C. rhomboidea*. Utilizing this variation to uncover trade-offs and mechanisms that may be driving drought resistance will help not only to pinpoint what drives cavitation vulnerability but also to determine the adaptive capacity of species, informing our efforts to predict and mitigate tree damage in the face of extreme climatic conditions.

## Supplementary Material

Supplementary_information_revised_tpad037

## Data Availability

The data that support the findings of this study are available from the corresponding authors upon reasonable request.
