# Supplementary material for: Evidence for a trade-off between growth rate and xylem cavitation resistance in Callitris rhomboidea
Source: Tree Physiol. 2023 Mar 22;43(7):1055–65. doi: 10.1093/treephys/tpad037 (PMC10335850; doi:10.1093/treephys/tpad037)
Supplement: Supplementary_information_revised_tpad037 [file supplementary_information_revised_tpad037.docx]

## Supplementary information

______________________________________________

**Figure S1**: Average P50s for internodes based on distance to the branch tip.

**Figure S2:** P50 (MPa) against Internode length (mm) for all internodes where length was measured.

**Figure S3:** Average tracheid area in µm^2^ (a) and diameters in µm (b) against distal branchlet internode length calculated from transverse light microscope cross sections of short (4mm or <) and long (8mm or >) distal branchlet internodes.

**Figure S4:** The density of margo papillae found through SEM imaging of pits in internodes for which vulnerability to xylem cavitation (P50) had been quantified, along with representative images of these pits, showing each of the four papillae density categories.

**Figure S5:** The average pit aperture area in µm^2^ calculated from SEM preparations of distal branchlet internodes with known P50s.

**Figure S6:** The percentage internode shrinkage (based on the initial, hydrated internode width) at -3 MPa and -5 MPa in both short (4mm or <) and long (8mm or >) distal branchlet internodes.

______________________________________________

**
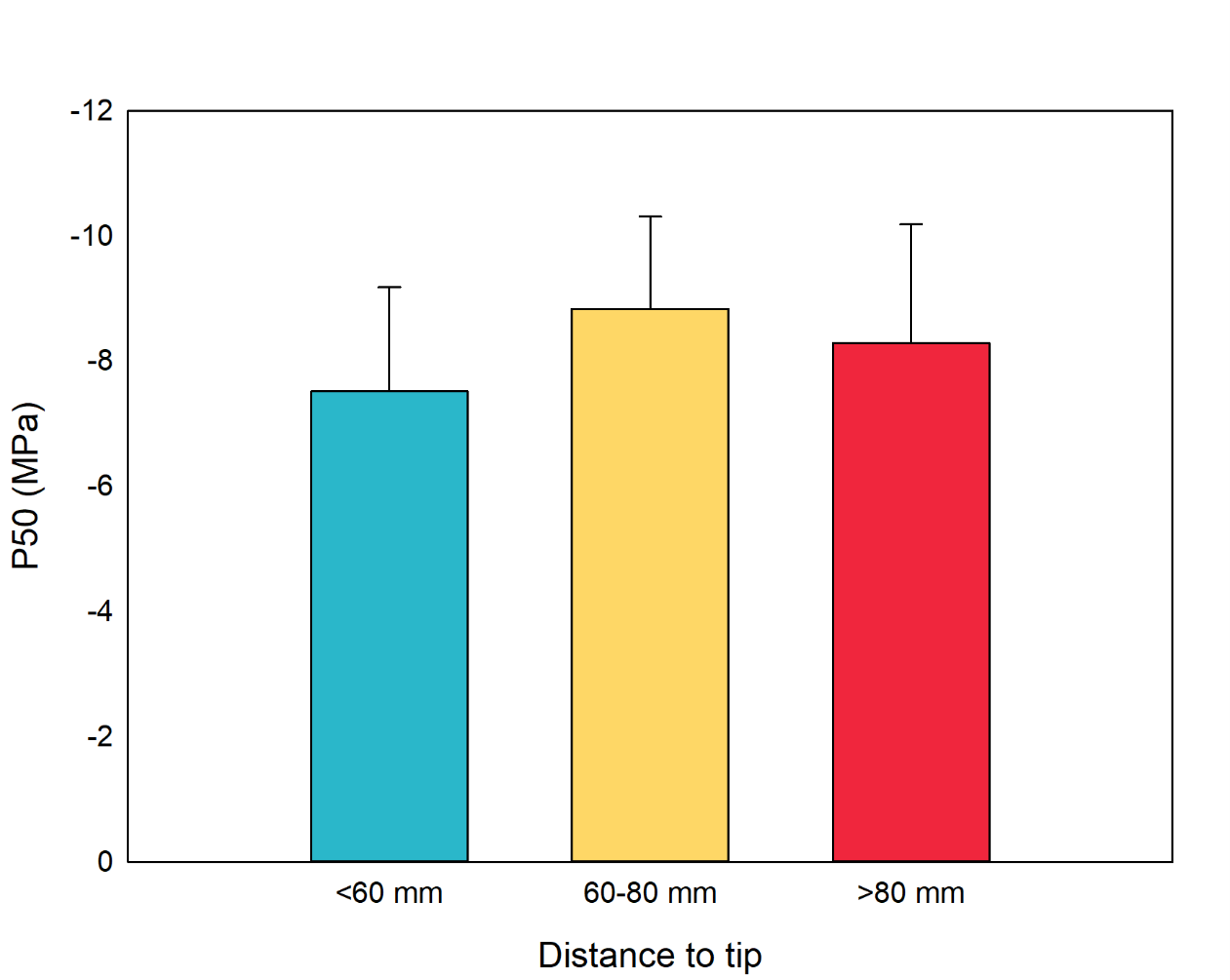
**

**Figure S1:** The average P50s for distal branchlet internodes within < 60 mm (blue), 60-80 mm (yellow) and > 80 mm (red) of the branch tip, showing no significant difference in the P50 across these groups (ANOVA;P> 0.05). The error bars show the standard error.


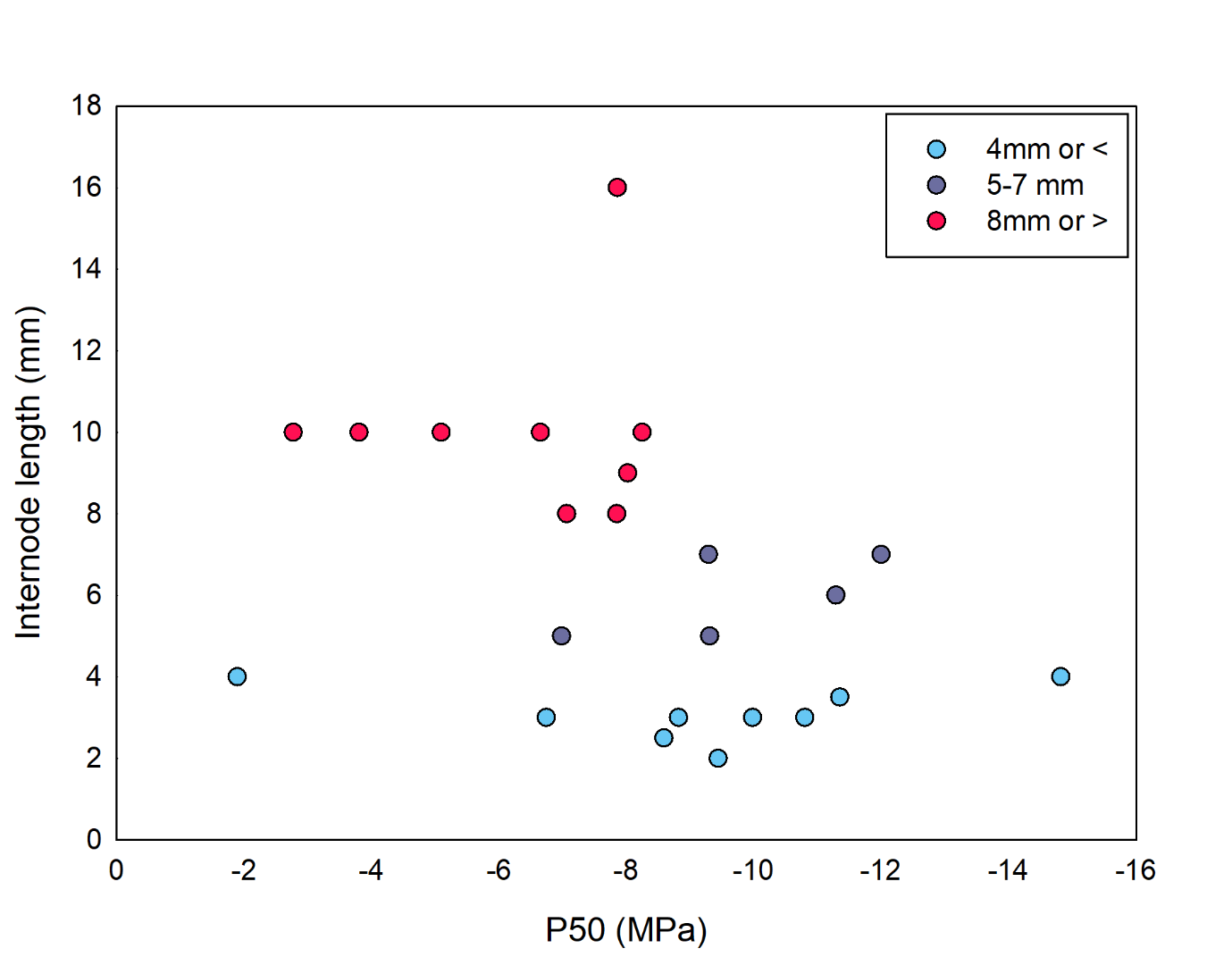


**Figure S2:** P50 (MPa) against Internode length (mm) for all internodes for which length was measured.


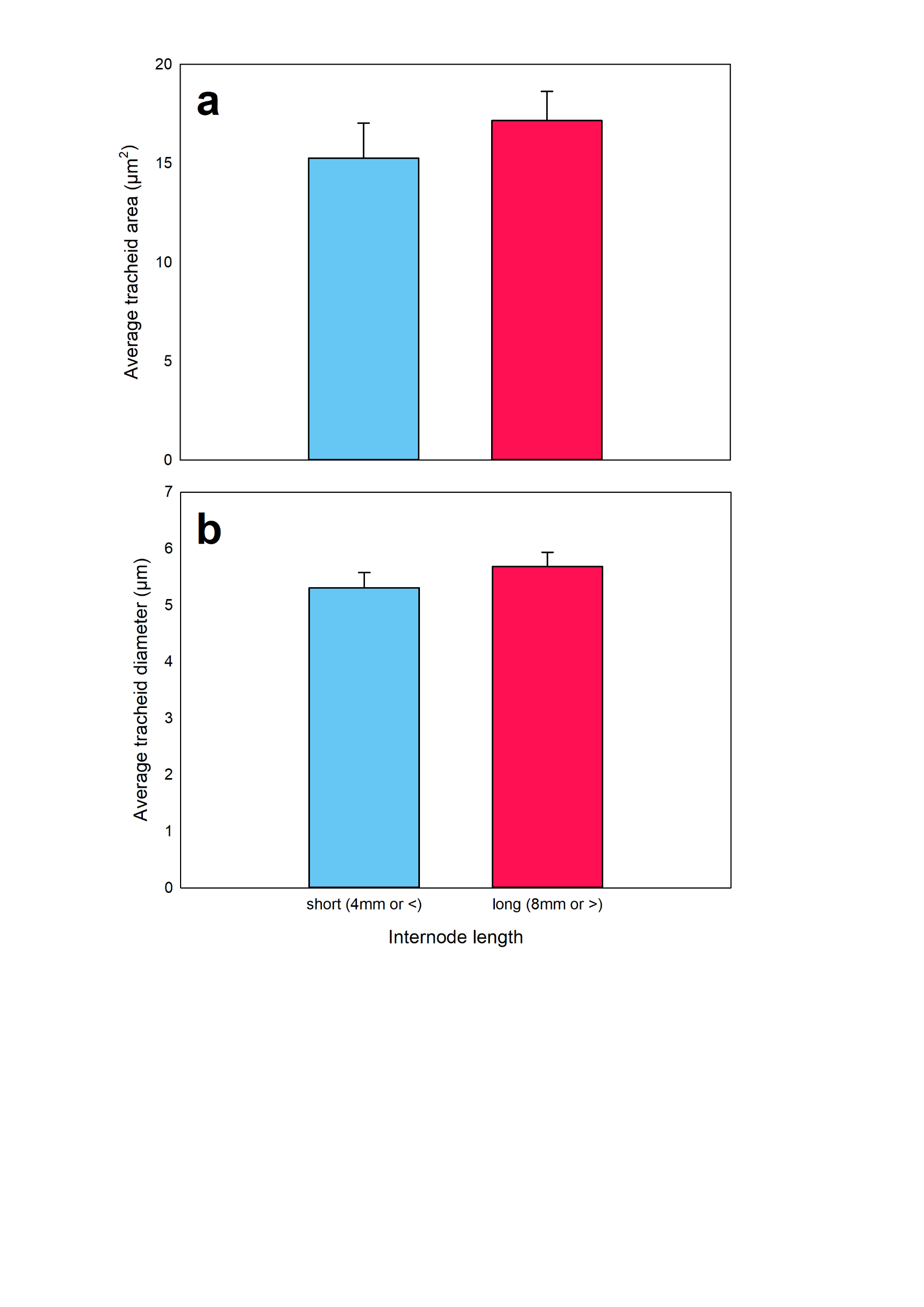
**Figure S3:** Average tracheid area in µm^2^ (a) and diameters in µm (b) against distal branchlet internode length calculated from transverse light microscope cross sections of short (4mm or <) and long (8mm or >) branchlet internodes**.** The error bars show the standard error.


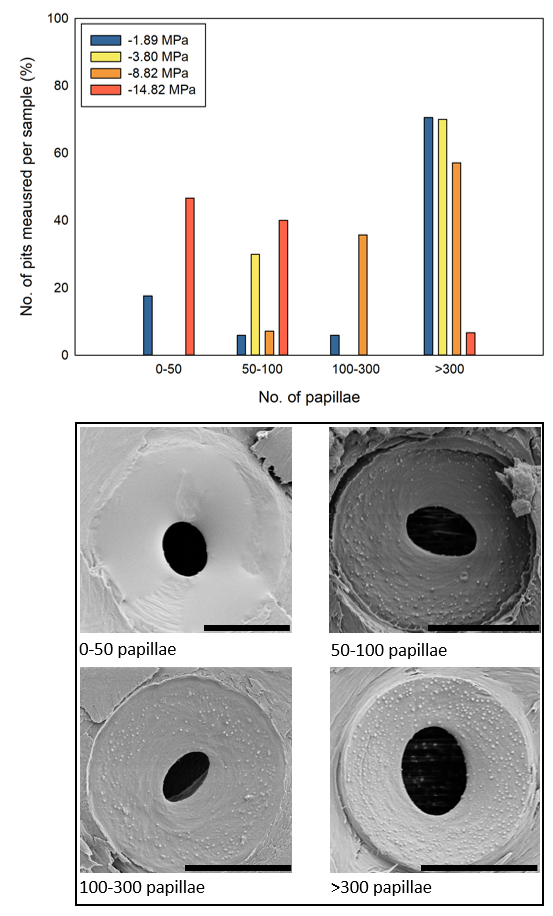


**Figure S4:** The graph (top) shows the density of margo papillae found through SEM imaging of pits in internodes for which vulnerability to xylem cavitation (P50) had been quantified (blue: -1.89 MPa, yellow: -3.80 MPa, orange -8.82 MPa and red -14.82 MPa)**,** along with representative images of these pits, showing each of the four papillae densities (bottom). The black scale bars at the bottom of each of these images represents 5 µm.


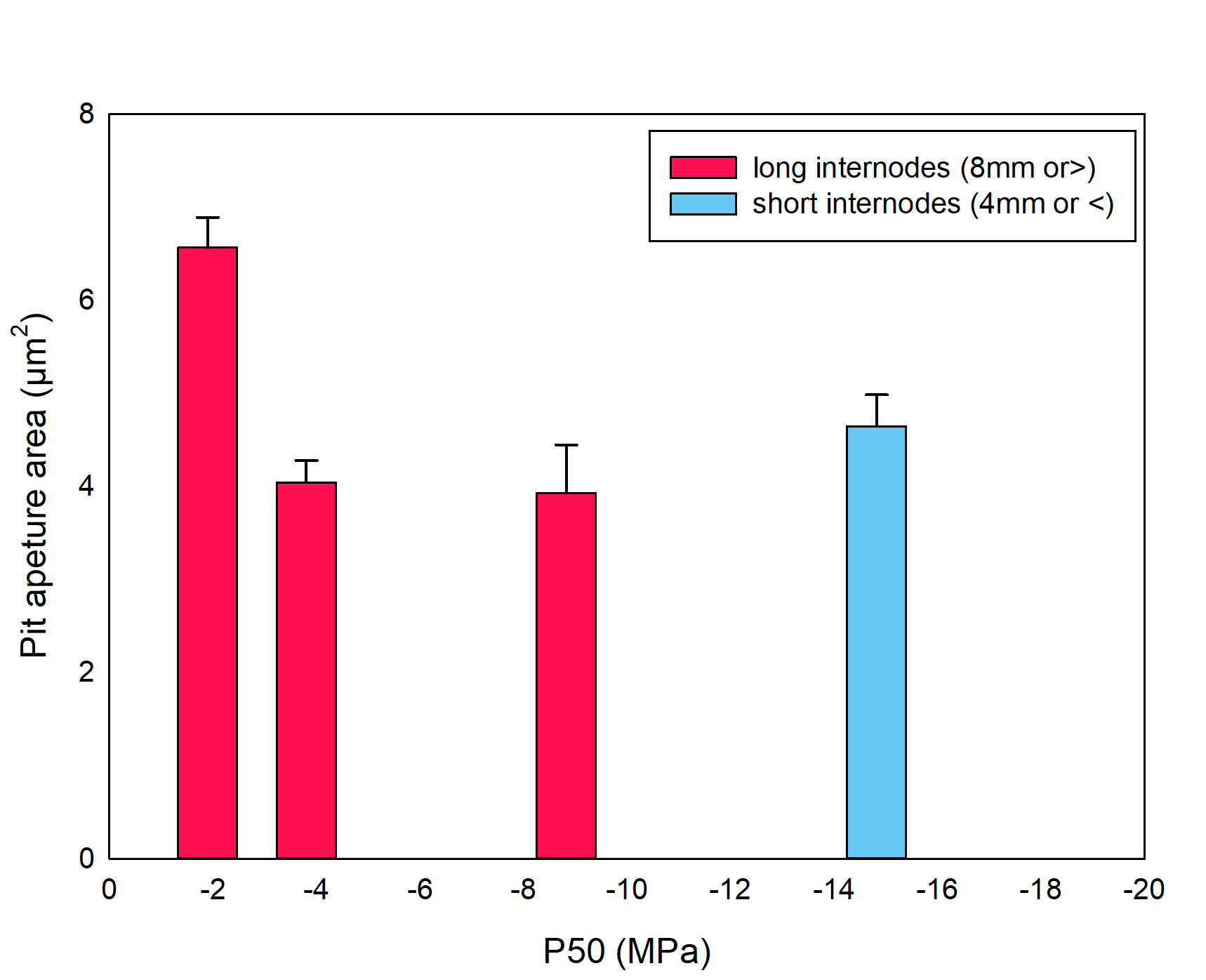
**Figure S5:** The average pit aperture area in µm^2^ calculated from SEM preparations of distal branchlet internodes with know P50s (-1.89 MPa, -3.80 MPa, -8.82 MPa and -14.82 MPa), where internodes are colour coded according to length (pink: long internodes 8mm or > and blue, short internodes 4mm or <). The error bars show the standard error.

**
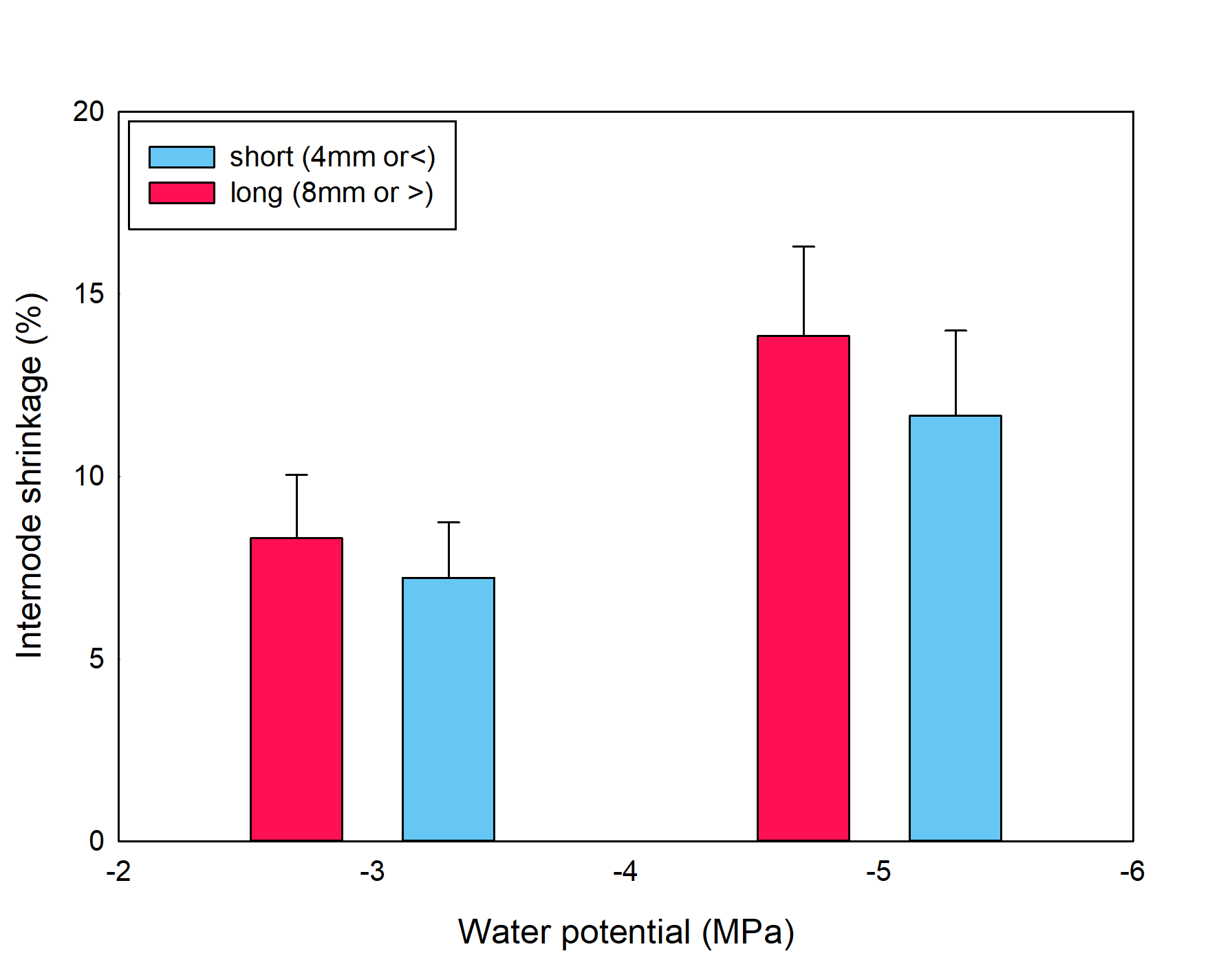
**

**Figure S6:** The percentage internode shrinkage (based on the initial, hydrated internode width) at -3 MPa and -5 MPa in both short (4mm or <) and long (8mm or >) distal branchlet internodes, where internodes are colour coded according to length (pink: long internodes 8mm or > and blue, short internodes 4mm or <). The error bars show the standard error.
